# Determining the prices of the medicines in the absence of superiority over alternative medical technology

**DOI:** 10.1186/2052-3211-8-S1-P11

**Published:** 2015-10-05

**Authors:** Wojciech Matusewicz, Katarzyna Jagodzińska-Kalinowska, Aneta Lipińska, Maciej Pomorski

**Affiliations:** 1 Agency for Health Technology Assessment and Tariff System (AHTATS), Warsaw, 02-611, Poland

## Problem statement

When there are no RCTs which prove that the new technology is better than the current drug or non-drug medical technologies currently financed by the public payer, the MAH is obliged to use the price equal to the cheapest reimbursed alternative.

## Objectives

Presentation of a method offered by the new Reimbursement Act (RA), which allows one to determine the prices of medicines in the absence of superiority over alternative medical technology. This price is calculated by the AHTATS and must be placed in the Recommendation of the President of the AHTATS.

## Methods

The primary goal of the new RA is to implement the principle of economical production. This should be kept in mind, while interpreting its content, because the act allows one to approach to each drug individually.

At the beginning one needs to find a comparator currently financed with the best outcomes/cost ratio. The next step is to search for RCTs proving the superiority of the proposed drug over the designated comparator. In cases, where there are no such RCTs in the MAH's submission, the official sales price of the drug, must be calculated in such a way that the cost of using it would not be higher than using the designated comparator. This has significant consequences because the Ministry of Health is obliged to use the calculated price in its final reimbursement decision.

## Results

The first example is a poly-pill compared with the separate tablets of the same substances. Probably there will be no studies that demonstrate the superiority of the poly-pill. Thanks to the RA, the cost of therapy with the poly-pill proposed by the applicant cannot be higher than the cost of therapy with the same substances in separate tablets. The second example is an add-on therapy. If there is no RCT proving the superiority, the price can be proportionally calculated as a share of the total cost of the treatment, which will be equal to the cost of the treatment with the exception of add-on therapy.

## Conclusions

The new RA offers the tool to set the maximum price, for the drugs without proven superiority, by comparison with the cost of the cheapest comparator or a comparator with the best ratio. This price should be the starting point in negotiations from the payer's perspective. Thanks to this price, presented in the Recommendation of the President of the AHTATS, the Ministry of Health in Poland has a better negotiating position.

